# Supplementary material for: Are carbon emissions trading and green financial instruments synergistic? -Comprehensive quantitative research based on content analysis
Source: PLoS One. 2024 Mar 7;19(3):e0298601. doi: 10.1371/journal.pone.0298601 (PMC10919601; doi:10.1371/journal.pone.0298601)
Supplement: S1 Data — (ZIP) [file pone.0298601.s001.zip › Data/地级市Z_F工作报告.zip!/╣π╬≈/╬α╓▌╩╨/└┤╫╘╣1⁄2╓┌║┼í╛┬φ┐╦╩2╛▌═°í┐_2016.docx]

各位代表：

　　现在，我代表市人民政府向大会作政府工作报告，请予审议，并请市政协各位委员提出意见。

一、2015年和“十二五”时期工作回顾

刚刚过去的一年，面对经济持续下行的压力，在自治区党委、政府和市委的正确领导下，在市人大、市政协的监督支持下，市政府团结和依靠全市人民，抢抓机遇，沉着应对，奋力拼搏，坚持以向东开放和改革创新为动力，全力抓好稳增长、促改革、调结构、惠民生、防风险各项工作，完成了市十三届人大六次会议和“十二五”规划确定的主要目标任务。初步核算，全市地区生产总值增长8.3%，规模以上工业增加值增长 8.6 %，固定资产投资增长19.3%，财政收入增长1.1%，社会消费品零售总额增长11.2%，城镇居民人均可支配收入、农民人均纯收入分别增长6.7%和8.5%。

一年来，我们重点抓了七个方面工作：

　　抓项目建设扩投资。坚持以“三年一工程”为抓手，大力推进西江经济带基础设施建设大会战、城市建设三年提升工程和“两区一城”建设，发挥了投资对稳增长的“压舱石”作用。全年安排重大项目464项，完成投资397亿元，占年度计划的102%。其中一批重大交通项目取得新突破，梧州至柳州高速公路、市环城高速公路、赤水铁路专用线等项目进展顺利，梧州至贵港高速公路、龙圩至藤县潭东一级公路建成通车，梧州南站至西江大桥路段等道路改造提升工程完工。

抓转型发展调结构。大力改造提升传统产业，全市完成更新改造投资447.2亿元，增长17.5%。积极培育高新技术产业，现有自治区级千亿元产业研发中心2家、自治区级产业化工程院3家、国家高新技术企业21家。加快推进梧州生态产业园、中节能环保产业园、静脉产业园、稀土产业园等重大项目，新兴产业逐步走向规模化、产业化。发展壮大农业特色优势产业，稳步推进7个特色农业示范区建设，其中有2个获得自治区级示范区称号。培育新型农业经营主体，新增市级农业产业化重点龙头企业9家，新成立各类农民专业合作社254家，新发展家庭农场82家。林下经济产值突破70亿元，蜂蜜产量位居全区第1位。实施加快第三产业发展系列措施，组织实施三产重点项目46个，开展系列大型促销活动，三产增加值增长10.6%，高出二产2.5个百分点，创下“十二五”以来的新高。

抓服务企业稳工业。继续实施工业巩固提升工程，落实降电价、减负担等政策措施，帮助再生不锈钢制品骨干生产企业争取到优惠电价政策，挽回产值超过200亿元。着力破解企业融资难问题，推选15家企业到区域性股权交易市场挂牌上市，成立融资担保公司，累计投入财政性资金6800万元用于开展“惠企贷”业务，帮助28家企业获得贷款2亿多元。组织开展“千名干部进千企”活动，帮助企业解决各类具体事项178个，全市共争取到各类企业扶持资金1.55亿元，园区发展资金3800万元，有力促进了工业经济运行平稳。全市规模以上工业总产值首次突破2000亿元，达到2141.3亿元，增长11.4%，成为稳增长的中流砥柱。

抓改革创新促开放。深入推进简政放权，市本级共调整、取消和下放行政审批项目160项，完成了第一批14个主要部门权力清单和责任清单的清理审核工作，商事制度改革不断深化，行政审批局和行政执法局筹建工作进展顺利。积极参与珠江—西江经济带建设，同广州签订了战略合作协议，承办了两广推进珠西经济带建设现场对接会。深化粤桂合作特别试验区体制机制改革创新，试验区联合管委会正式挂牌成立，梧肇双方实现合署办公，开发建设全面提速。试验区入驻企业总数达181家，其中梧州片区125家；新开工产业项目25个，建成投产企业15家。梧州口岸赤水作业区扩大开放获国务院批复同意，临港经济区加快建设。成功举办第十二届梧州宝石节、第八届西江经济发展论坛、第二届中国（梧州）健康养生产业资源合作与投资促进大会、第二届广西（梧州）粤剧节等重大节庆活动。

抓城乡统筹优生态。大力推进城市建设三年提升工程各类项目154个，完成投资超过70亿元。城市总体规划修编完成并获自治区批复。成功承办广西第十三届运动会和第六届广西园林园艺博览会，建成体育场、游泳（跳水）馆、综合馆、网球中心等体育场馆，园博园成为我市热门旅游景点。承办了全区棚户区改造工作现场会，全年棚改开工22000多套（户）。继续推进农村危房改造，完成了13500户改造任务。积极开展生态文明示范区创建工作，全市有5个镇和25个村分别获得“自治区级生态乡镇”、“自治区级生态村”称号，蒙山县被列为国家级生态功能区并获得“全国休闲农业与乡村旅游示范县”称号。整合资金5亿多元投入生态乡村建设，农村生态环境进一步改善。

抓民生改善保稳定。全年财政支出突破200亿元，增长16.3%，较好地实现了保民生、保重点、保运转的目标。切实办好12项民生实事，完成投资45亿元。持续推进“安居乐业”工程，基本建成保障性住房6619套，分配入住5677套；全市城乡新增就业7.7万人次，城镇登记失业率控制在4.5%以内。义务教育发展取得新成效，万秀区通过自治区义务教育均衡发展评估验收。医药卫生和养生养老工作取得新进展，我市被全国老龄办确定为全国老年友好型城市创建试点市，荣获“全国基层中医药工作先进单位”称号。全力推进精准扶贫、精准脱贫，全年脱贫人口4.5万人。扎实推进“平安梧州”建设，应急管理工作扎实有序开展，食品药品安全监管能力不断提升，刑事案件、治安案件及安全生产事故四项主要指标均比上年下降，社会保持和谐稳定。

抓作风建设提效能。深入开展“三严三实”专题教育，贯彻落实中央“八项规定”精神，积极推进公车改革，市本级公共财政安排的“三公”经费支出下降6.4%，会议费用下降 20.1%，全年政府采购节约率为7.4%。提升政务服务水平，积极推动市本级行政审批职能部门集中办理审批业务，全市997个行政村（社区）均建立政务服务中心，基层政务服务覆盖率达到100%。加快建设法治政府，抓好重大项目法律顾问服务工作，将依法行政工作纳入绩效考核，推动政府重大事项集体决策、科学决策、民主决策。圆满完成“六五”普法工作。自觉接受人大及其常委会法律监督和工作监督，主动接受政协民主监督，人大代表建议和政协提案办结率100%。

一年来，国防建设、国防动员、民兵预备役、人民防空及双拥工作得到加强，编制、审计、民族、宗教、外事侨务、对台、统计、物价、国资、供销、农机、地震、移民等工作取得了新进展，工会、共青团、妇女儿童、残联、科协、社会科学、慈善、档案、地方志等工作取得新进步，中直、区直驻梧各单位为全市经济社会发展作出了积极贡献。

2015年，我市经济社会发展实现了稳中有进、稳中提质、稳中有好，为“十二五”划上了圆满句号，为“十三五”发展打下了坚实基础。

五年加快发展，综合实力显著提升。“十二五”期间，全市地区生产总值由579亿元迈上1000亿元台阶，年均增长11%；人均地区生产总值由1.9万元增加到3.6万元，总体进入中等收入阶段；财政收入由56.1亿元增加到123.7亿元，年均增长17.1%；社会消费品零售总额由191.8亿元增加到364.9亿元，年均增长13.7%。

五年转型发展，产业结构加速优化。三次产业结构由2010年的14：59：27调整为2015年的11：58：31，三产比重提高了4个百分点。产值超百亿元的园区增至7个。以再生资源、再生不锈钢制品产业为主体的循环经济产业年产值接近900亿元。旅游产业取得新成效，全市接待旅客总人数和旅游收入年均分别增长17.8%、23.9%。

五年优先发展，交通基础不断夯实。“十二五”期间，全市完成全社会固定资产投资4328.9亿元，其中交通基础设施投资240亿元。梧州迈入高铁时代，高铁营运里程达89公里，群众出行更加便利。新增公路里程821.5公里，其中新增高速公路里程57公里、总里程达275公里。新增2000吨以上级泊位14个，港口吞吐能力达5000万吨。西江机场开工建设。梧州作为区域性综合交通运输枢纽的地位进一步提升，区位优势进一步显现。

五年向东开放，发展活力持续增强。粤桂合作特别试验区建设纳入《珠江—西江经济带发展规划》，上升到国家战略层面，成为两广战略合作的标志性工程。区域合作取得新突破，梧州成为广西首个与广州、佛山、肇庆、云浮4个广东珠西经济带城市开展全面战略合作的城市。“十二五”期间，我市累计引进外来投资项目1928个，招商引资到位资金2883.4亿元，其中60%以上来自粤港澳地区。

五年拓展提升，城乡面貌明显改善。城镇化进程明显加快，行政区划调整如期完成，中心城区建成区面积拓展到90平方公里，全市城镇化率达52%。红岭新区功能日趋完善，苍海新区基本实现“成湖成景”的阶段性目标，苍海湿地公园获批为国家湿地公园试点。无障碍环境建设取得明显成效，我市被评为全国无障碍建设示范市。三县一市的县城面貌明显改观，建成了一批美丽宜居乡镇、生态乡镇和生态村，蒙山县永安王城被评为国家4A级旅游景区。生态环境持续改善，全市森林覆盖率达75.8%，荣获“国家森林城市”、“国家园林城市”称号，进一步打响了“山清水秀生态美”的城市品牌。

五年聚力民生，社会事业全面进步。财政民生支出占比逐年提高，2015年达到80%以上。城镇居民人均可支配收入、农民人均纯收入年均分别增长9.5%、13.2%。学前三年毛入园率达76%，九年义务教育巩固率达94%以上，高中阶段毛入学率达87%。我市在广西第十三届运动会上取得优良成绩，全民健身氛围日益浓厚。覆盖城乡的社会保障体系、城乡医疗服务体系和公共文化服务网络体系基本形成。全市减少贫困人口24.6万人，完成民生水利投资65亿元，解决了89.3万农村人口饮水安全问题，城乡新增就业38.5万人次。

各位代表，过去五年取得的成就，是自治区党委、政府和市委正确领导的结果，是市人大和市政协监督支持的结果，是全市340万人民团结拼搏的结果。在此，我代表市人民政府，向全市各条战线的干部群众，向人大代表、政协委员，向各民主党派、工商联、无党派人士、人民团体，向离退休老领导、老同志，向驻梧部队、武警官兵、公安干警，向所有关心支持梧州发展的社会各界人士，表示崇高的敬意和衷心的感谢！

回顾五年工作，我们也清醒地认识到，我市后发展欠发达的基本市情还没有根本改变，经济社会发展仍面临一些困难和问题。主要是：经济总量小，人均水平不高，区域比较优势有待充分发挥；自主创新能力不足，先进制造业和现代服务业发展滞后，第三产业比重偏低，转方式调结构任务艰巨；基本公共服务有待加强，城乡发展不平衡，城乡居民持续增收困难加大；全方位开放水平有待提高，加快发展面临一些体制机制障碍，行政审批效率有待进一步提高，等等。这些问题和不足，我们将高度重视，在今后的工作中采取有效措施认真加以解决。

二、“十三五”时期的奋斗目标和主要任务

市委十二届六次全会提出，今后五年我市经济社会发展的总体目标是“三个实现”：即实现与全国和全区同步全面建成小康社会，实现基本建成西江黄金水道上向东开放的龙头城市，实现基本建成布局优化、生态宜居的桂东南城镇群核心城市。我们要抢抓国家建设“一带一路”、打造中国—东盟自贸区升级版、深入实施珠西经济带建设规划，以及自治区推进“双核驱动”战略、加快构建两广经济一体化等重大机遇，切实增强历史使命感和责任感，敢于担当，勇于拼搏，勇于创新，努力走出新常态下具有梧州特色的发展之路。

“十三五”时期，我市经济社会发展的总体要求是：高举中国特色社会主义伟大旗帜，全面贯彻党的十八大和十八届三中、四中、五中全会精神，以马克思列宁主义、毛泽东思想、邓小平理论、“三个代表”重要思想、科学发展观为指导，深入贯彻习近平总书记系列重要讲话精神，按照“四个全面”战略布局，坚持发展第一要务，牢固树立创新、协调、绿色、开放、共享的发展理念，注重转型升级和特色效益发展，深度融入珠三角，主动对接北部湾，以实施开放、创新战略为主线，以深化改革为动力，加快形成引领经济发展新常态的体制机制和发展方式，统筹推进经济建设、政治建设、文化建设、社会建设和生态文明建设。

围绕上述目标要求，市政府根据《中共梧州市委员会关于制定国民经济和社会发展第十三个五年规划的建议》，在广泛征求各方意见的基础上，制定了《梧州市国民经济和社会发展第十三个五年规划纲要（草案）》。《纲要（草案）》共分3大板块13篇47章。第一板块由第一篇构成，分析“十三五”发展环境，明确指导思想、基本原则、发展目标和发展理念。第二板块由第二篇至第十二篇构成，安排部署经济社会发展的主要任务和各项工作。第三板块由第十三篇构成，提出保障措施。《纲要（草案）》已印发，请各位代表连同《报告》一并审议，我在这里着重报告三个方面内容：

（一）关于“十三五”主要指标。

与全国全区同步全面建成小康社会，是我市“十三五”期间最硬的任务。全面建成小康社会，包括经济发展、创新驱动、民生福祉、生态文明等四大类33项具体指标，其中最关键的就是确保到2020年实现地区生产总值和城乡居民收入比2010年翻一番以上，基于此，我市未来五年地区生产总值和城乡居民收入年均增长设定为8%以上。提出这一目标，综合考虑了三个原则：

一是体现助跑摸高原则。根据测算，由于“十二五”打下的良好基础，我市地区生产总值和城乡居民收入分别只需保持年均增长3.5%和7.7%，就可以实现翻一番的目标。然而，综合研判“十三五”时期发展环境，欠发达后发展仍是我市基本市情，赶超跨越仍是长期而艰巨的任务。只有设置高于全国全区平均水平的经济增速，增强责任意识和忧患意识，抢抓机遇，加快发展，才能赶上全国发展步伐，努力走在全区的前列。

二是体现脚踏实地原则。尽管我们面临经济持续下行的压力，但上述两项指标实现年均8%以上增长目标，是有基础、有条件、有支撑的。从发展态势看，近年来我市经济增速一直高于全国全区平均水平，只要保持良好势头，目标完全可以实现。从支撑条件看，第一产业需年均增长4.0%，第二产业需年均增长7.8%，第三产业需年均增长11%，据测算，经过努力都能实现。从收入水平看，随着一系列惠民政策的落实和收入倍增计划的实施，我市城乡居民收入将保持较快增长，实现年均8%以上的增长目标也完全有条件。

三是体现务求实效原则。经济发展步入新常态，核心就是速度换挡、动力转换、发展转型。保持高于全国1.5个百分点、全区0.5个百分点的中高速增长，既可以保证换挡不失速，保住就业、收入、民生等底线，又为转方式、调结构、补短板腾出空间。经济由“量变”转向“质变”，并不是放弃经济增长，而是把速度保持在合理区间，把质量放到首位，通过位置调换和工作重心调整，让经济增长更有含金量，让人民群众共享发展成果。

（二）关于“十三五”主要考虑。

实现“十三五”时期发展目标，必须结合我市的战略定位和特色优势，牢固树立创新、协调、绿色、开放、共享的发展理念，注重转型升级和特色效益发展，促进发展方式由规模速度型向质量效益型转变。

更加注重创新驱动。围绕我市产业发展需要，强化产学研合作，打造一批具有全国竞争力的创新型领军企业。推进创新创业平台建设。实施人才小高地建设提升工程，大力发展“互联网+”创新创业网络体系，推动大众创业、万众创新。进一步转变政府职能，大力推进重点领域改革，积极营造创新发展环境和建立创新发展机制。从供给和需求两侧发力推动结构性改革，增强发展新动力。通过五年努力，科技进步贡献率达到55%，科研经费占地区生产总值比重提高到2.1%，建成自治区级自主创新示范区，积极创建国家级自主创新示范区。

更加注重开放带动。以东向开放带动全面开放，主动融入“一带一路”，全面落实珠西经济带发展规划，积极参与粤桂黔高铁经济带建设，深化区域城市战略合作，全面加快粤桂合作特别试验区建设，全方位、多角度打造粤桂深度合作前沿阵地。推动外贸向优质优价、优进优出转变，支持优势企业参与境外产能合作，大力培育跨境电子商务、市场采购贸易等新型贸易业态，提升开放型经济发展水平。通过五年努力，打造珠西经济带新增长极，加快形成全方位开放合作新格局。

更加注重协调发展。全面落实“五位一体”发展总体布局，补齐全面建成小康社会短板，缩小城乡居民收入差距。继续实施城市建设提升工程，加强城镇基础设施建设，推动市区浔江南岸片区开发建设，实施长洲岛综合开发整治工程。推进大县城和特色镇发展战略，有序推进农业转移人口市民化，构建“一带两区三走廊”的城镇化发展格局。通过五年努力，市区建成区面积达到120平方公里，常住人口118万人，基本建成桂东南城镇群核心城市。

更加注重绿色发展。强化主体功能定位和国土开发强度“双约束”，推进多污染物综合防治和环境治理，实行联防联控和流域共治，所有县城和建制镇污水垃圾处理设施实现全覆盖，市区基本实现污水垃圾全收集全处理，县城生活污水集中处理率达到85%。深入推进“美丽梧州·生态乡村”工作，加快向宜居乡村、幸福乡村迈进。大力发展循环经济，促进各类产业园区低碳循环发展，建设全国循环经济示范城市。通过五年努力，主体功能区布局基本形成，生态经济体系基本建成。

更加注重共享发展。切实把保障和改善民生摆上更加突出位置，持续加大民生投入，实施重点领域民生工程，健全社会保障体系，深化收入分配改革，更加注重对特定人群、特殊困难群体的精准帮扶。推进法治建设，创新社会治理，建设社会信用体系，维护社会公平正义。通过五年努力，实现人民生活显著改善，公共服务体系更加健全，贫困现象得到消除，同全国全区同步迈入全面小康社会！

（三）关于“十三五”主要任务。

针对我市经济社会发展的主要瓶颈和薄弱环节，突出抓好产业发展、基础设施、公共服务和改善民生等主要任务。

构建竞争力较强的特色现代产业体系。全面推进产业转型升级，推动产业结构从中低端向中高端跨越，让传统产业迸发活力，让新兴产业形成新动能。

——改造提升现有优势工业。推动现有优势产业提高附加值、延伸产业链，打造千亿元、五百亿元、百亿元产业集群，推动工业迈向中高端水平。大力实施“互联网+工业”行动，促进工业化和信息化融合。工业增加值达到770亿元，年均增长8%以上。

——培育壮大新兴产业。深度承接产业转移，加快建设先进制造业基地，培育壮大战略性新兴产业，力争新兴产业增加值占地区生产总值比重达到15%以上。

——增强园区综合承载能力。大力创新园区管理体制和运行机制，激发园区发展活力，提升园区经济整体竞争力，实现工业园区产值占全部工业总产值90%以上。

——提速发展现代服务业。推动生产性服务业与制造业相融合，推进生活性服务业升级发展，建成一批自治区级现代服务业集聚区，服务业增加值比重提高到42%，社会消费品零售总额年均增长10%以上。

——大力发展现代农业。稳定粮食播种面积和综合生产能力。培育发展优势种养业，加快发展林下经济，重点打造百亿元六堡茶产业集群，建设面向珠三角和港澳地区的优质农副产品供应基地。

构建比较完善的基础设施体系。全面推进基础设施建设攻坚战，围绕做强枢纽、优化网络、提升保障目标，构建功能配套齐全、高效安全便捷的现代基础设施体系。

——完善公路网络。基本建成“一环七射三连线”高速公路网络，基本实现县县通高速、村屯通硬化路。争取全市公路总里程突破6800公里，其中高速公路通车里程500公里以上。

——完善铁路网络。建成岑溪至罗定铁路和赤水铁路专用线，推动柳州经梧州至广州铁路、柳州经梧州至韶关铁路、洛阳至湛江铁路梧州段电气化扩能改造开工建设，争取铁路通车里程突破400公里，形成“多线放射”的铁路网络。

——完善水运体系。加快推进西江、桂江干流航道整治工程，建成一批港口码头，实现梧州港口吞吐能力达6000万吨，集装箱吞吐能力150万标准箱。

——建设桂东南航空枢纽。建成梧州西江机场及通用航空基地，开工建设岑溪、蒙山通用机场，推进苍海、苍梧通用机场前期工作。积极拓展航线，拓宽通航业务，形成辐射桂东粤西的区域性干线机场。

——提升水利保障能力。建设一批水利设施重点项目，实施农村饮水巩固提升工程，建设水生态廊道体系，争创国家海绵城市试点、国家水生态文明试点城市。

——提升能源支撑能力。积极推进新型电源项目建设，加快推进覆盖城乡的天然气输送网络，建设国家新能源示范城市。

构建均等化的公共服务保障体系。牢固树立共享发展理念，坚持以人为本，保基本、兜底线、促公平，健全基本公共服务体系，实现全民共同迈进全面小康社会。

——提升教育发展水平。加大教育经费投入，推进基础教育均衡发展，大力发展现代职业教育，提升高等教育质量。到2020年，全面解决幼儿入园难问题，实现县域义务教育均衡发展，基本普及高中教育，中等职业教育在校生规模达到6万人。

——推进健康梧州建设。实施健康产业重点工程，建立覆盖城乡的基本医疗卫生体系，推动医疗服务县乡一体化，从源头上保障食品药品安全，发展多层次养老服务，人均预期寿命提高到77.8岁以上。

——建成覆盖城乡的公共文化服务体系。实施文化惠民工程和重大公共文化工程，推动公益性文化设施免费开放。大力发展体育事业和体育产业。深入发掘岭南文化资源，保护历史文化遗产，恢复重建两广总督府等历史建筑，申报创建国家历史文化名城。

大力增进民生福祉。持之以恒保障和改善民生，为百姓创造更多的福祉，让人民群众有更多获得感。

——实现更加充分、更高质量的就业。实施更加积极的就业创业政策，统筹推进农村转移劳动力、城乡就业困难人员就业。城镇新增就业人数累计超过10万人，城镇调查失业率控制在6%以内。

——持续增加城乡居民收入。深入实施城乡居民收入倍增计划，健全科学的工资水平决定机制、正常增长机制、支付保障机制，多渠道促进居民增收。提高农民职业技能和创收能力，千方百计增加农民收入。

——建立更加公平可持续性的社会保障制度。实施全民参保计划，建立公平统一规范的城乡居民养老保险制度，全面实施城乡居民大病保险制度，完善失业、工伤、生育保险制度。健全社会救助体系，完善城乡最低生活保障制度。

——全面推进精准扶贫精准脱贫。这是我市“十三五”时期最大的政治责任、最大的民生工程，必须坚决打赢脱贫攻坚战，实现贫困县、贫困村全部摘帽，现行标准下的23.14万农村贫困人口全部脱贫。

各位代表！蓝图已经绘就，实干托起梦想。我们坚信，只要全市各族人民万众一心、努力拼搏，“十三五”的奋斗目标一定能如期实现，梧州的明天一定会更加美好！

三、2016年的主要目标和重点工作

今年是“十三五”的开局之年，也是推进结构性改革的攻坚之年。我市经济社会发展的主要预期目标是：地区生产总值增长8%，固定资产投资增长14%，规模以上工业增加值增长8.6%，财政收入增长5%，社会消费品零售总额增长10%，外贸进出口总额增长12%，居民人均可支配收入增长10%，城镇登记失业率控制在4.5%以内，节能降碳减排等约束性指标达到自治区的要求。

实现上述目标，必须重点抓好以下工作：

（一）加大力度扩大有效投资。当前，投资仍然是拉动经济增长最直接、最有力的举措，必须发挥好有效投资对稳增长、调结构的关键作用。

抓好重大项目建设。全市统筹安排重大项目467个，年度投资420亿元以上，其中新开工90个以上、续建245个、前期132个。交通方面，加快推进西江机场、市环城高速公路、梧州至柳州高速公路等一批在建项目，建成赤水铁路专用线、岑溪至水汶高速公路，推动岑溪至罗定铁路复工，力争新开工荔浦至玉林高速公路蒙山段、贺州至巴马高速公路蒙山段、梧州至藤县藤州镇二级公路、国道355线苍梧梨埠至昭平马江二级公路等项目，加快信都至梧州高速公路、柳州经梧州至广州铁路、洛阳至湛江铁路梧州段扩能改造、柳州经梧州至韶关铁路等项目前期工作。水利方面，重点加快长洲南北堤、高旺防洪堤、平浪防洪堤、塘源防洪堤、藤县河东防洪堤、大壬灌区节水改造、苍海湖东岸防洪排涝综合整治工程等重点项目建设。电力能源方面，重点推进分布式能源发电、产业园区光伏发电等项目建设，加快500千伏章田输变电工程、岑溪天然气支线管道等项目前期工作。围绕自治区成立60周年大庆，积极争取自治区服务基层十大类民生项目。

加快推进项目落地。实施重大项目精细化管理，完善市领导联系重大项目责任制、项目联合督查工作机制、联合审批及问题协调会议制度，及时协调解决土地、资金、配套服务等问题。开辟项目审批“绿色通道”，清理规范各类中介服务，提高政府投资项目评审效率。继续实施重点项目审批代办服务，为项目建设提供全方位、全天候、全过程服务，促进项目早开工、早竣工。

充分激活民间投资。发挥政府投资的导向和杠杆作用，重点围绕基础设施、公共服务、民生工程等领域，向社会推出一批PPP项目，力争民间投资有较大幅度提升。积极对接自治区产业投资引导基金，力争募集政府投资基金50亿元以上。鼓励各类社会资本通过特许经营、政府购买服务、股权合作等多种方式参与项目建设及运营。鼓励和引导金融机构加大对优质企业的贷款支持。

（二）加大力度推动工业经济平稳发展。稳定工业主导地位，更加注重培育工业经济新的增长点，优化存量、提升增量、做大总量，提高工业发展质量和效益。

推动工业转型升级。继续实施一批重大技改项目，力争工业投资、技改投资均增长10%以上。推动现有企业提质增效，支持骨干企业做大做强，力争全市规模以上工业企业达400家、产值超亿元企业250家。大力培育新兴产业，加快新加坡产业园、国光电子产业园、中兴云数据中心及IT产业基地等新兴产业基地建设。大力发展生态经济，推动梧州生态产业园建设。实施“互联网+”改造传统产业，推动再生资源、食品、医药、陶瓷建材、人工宝石等传统产业与互联网融合发展，推动传统产业信息化、智能化转型升级。

提升服务企业水平。持续开展“千名干部进千企”帮扶活动，实行“一企一策”和 “一站式”服务，帮助企业解决项目建设和生产经营中遇到的问题，努力为企业发展创造良好环境。落实中央和自治区稳增长政策措施，清理和规范涉企行政事业性收费，帮助企业降低成本，切实为企业减轻负担。加大“惠企贷”实施力度，组建担保公司，推动企业进入资本市场直接融资，拓宽企业融资渠道。建立企业家交流培训平台，提升企业管理水平。实施农民工转移就业“订单培训”计划，为企业发展提供优质人力资源。

推进产业园区建设。完成临港经济区规划，加快基础设施建设，实现梧州口岸赤水作业区通关运行。启动梧州高新区二期建设，积极推进科技企业孵化器建设，加快国家级高新区创建工作。大力推动再生资源和再生不锈钢园区扩园提质，拉长再生铜、再生铝、再生塑料、再生不锈钢等产业链，力争循环经济产业产值突破1000亿元。促进岑溪市西部农民创业园、苍梧县旺甫工业小镇、蒙山县茧丝绸工业园区等县域产业园区加速成长。确保全市工业园区工业总产值突破2000亿元。

（三）加大力度推动服务业大发展。切实把加快服务业发展作为补短板的重点，创新发展模式，做大实体经济，加快调整优化产业结构。

大力发展商贸物流业。围绕建设广西现代服务业集聚区副中心城市的目标，加快西江综合物流园、市商贸物流园区、毅德商贸物流城等项目建设，打造以粤桂合作为核心的现代服务业集聚发展集中区。推动生产性服务业向专业化转变和价值高端延伸，重点加快粤桂合作特别试验区两广服务业合作示范区建设，力争引进培育若干实力雄厚、知名度较高的现代物流龙头企业。抓好梧州消费购物节、百店大促销、名特优农产品展销会等活动。力争今年社会消费品零售总额突破400亿元。

积极培育电子商务业态。实施电子商务“十百千万”工程，引进和培育知名电商企业和电商平台，鼓励个体商户利用第三方平台建立网上店铺。加快推进珠西经济带（梧州）跨境电商产业园、安富利电子产业园建设，努力打造区域性国际商品集散分拨中心和跨境电子商务服务基地。

加快发展旅游业。围绕发展城市旅游打造龙头景区，加快苍海旅游度假区建设，推动骑楼城—龙母庙、白云山—四恩寺、永安王城等景区景点提质升级，规划建设军事博览中心等一批旅游项目。整合优质旅游资源，推动旅游与文化等产业融合发展，大力发展休闲生态旅游，创建旅游名县名镇名村，扎实推进乡村旅游集聚区（带）建设和旅游精准扶贫工作。加强旅游宣传推介，重点吸引广西和粤港澳游客到梧州观光旅游、休闲养生。力争全市接待旅游总人数1660万人次、旅游总收入170亿元。

（四）加大力度抓好“三农”工作。围绕农业强、农村美、农民富目标，积极推动农业发展方式转变，努力走出一条集约、高效、安全、持续的现代农业发展道路。

加快发展现代农业。加强农业现代化基础建设，确保口粮安全。实施特色农业产业提升行动，推动特色种养业和休闲观光、都市农业、生态循环等新型农业融合发展。加快现代特色农业（核心）示范区建设，建成自治区级示范区3个、市级示范区3个。培育壮大多元经营主体，争取创建农民专业合作社示范社20个以上，新增市级农业产业化重点龙头企业5家以上。大力发展农村电子商务，培育“互联网+农业”新业态。

打好脱贫攻坚战。抓好全市265个贫困村的精准扶贫、精准脱贫工作，帮助移民库区、贫困村、贫困户发展特色富民产业，实现村有主导产业、户有增收门路，增强贫困村“造血”功能。推进贫困村水、电、路、网络等基础设施建设。力争全年减少贫困人口5.8万人，贫困人口人均纯收入增长15%以上。

深入推进生态乡村建设。实施改房、改水、改厕、改能、改路“五改”工程，不断改善农村人居环境和乡村面貌。把打造生态乡村活动示范点与休闲生态景区建设相结合，打造市、县、镇三级共282个生态乡村活动升级版示范点。

（五）加大力度推进新型城镇化。坚持协调发展，主动适应经济发展新要求和人民群众新期待，创新思维，加大投入，全面推进以人为核心的新型城镇化建设。

抓好中心城区建设。深入实施城市建设三年提升工程，力争完成投资80亿元以上。重点抓好静脉产业园、西江三桥、西江四桥等基础设施建设，完善城市公共服务设施，开展城市地下综合管廊建设试点，加快推进海绵城市建设。加快苍海新区建设，重点抓好主干路网、环城水系和高端商务、文化、旅游等项目建设。进一步完善红岭新区基础设施和公共服务设施，加快推进太和新城及市区浔江南岸片区开发建设，启动长洲岛综合整治工程。抓好棚户区改造，确保完成16267套（户）任务。

推进城乡一体化发展。高起点规划建设苍梧新县城，继续推进新县城主干道、苍梧中学石桥校区、公租房等项目建设，推动新县城给排水工程、妇幼保健院、文化公园等一批项目开工建设。推进岑溪新型城镇化试点县建设，推动藤县撤县改市，支持蒙山打造特色旅游名县。加快苍梧县旺甫镇、岑溪市归义镇、藤县太平镇及濛江镇、蒙山县新圩镇等中心镇发展，推动城镇市政设施向农村延伸、公共服务向农村覆盖，促进人才、技术、资金等生产要素投向农村。

加强生态环境建设。重点推进一批乡镇污水处理厂和垃圾片区处理中心建设。推进饮用水水源地整治工程，加快整治水源保护区内的污水直排口。加大对空气质量“PM10+PM2.5联合控制”力度。完成自治区下达的黄标车和老旧车淘汰任务。加强对排污企业的监督，严厉处罚偷排超排行为。加大市区宝石加工污染治理力度。深入实施造林绿化工程，完成植树造林18万亩，建设自治区级现代特色林业示范区。

（六）加大力度推动改革举措落地。改革创新是实现后发赶超的关键之举，必须抓好各项改革创新举措落地，不断增强发展的动力活力。

推动供给侧结构性改革。按照中央和自治区的部署，积极去产能去库存。去产能方面，重点是发挥好市场配置资源的作用，突出增强企业活力，推动企业通过更新改造、兼并重组等方式淘汰落后产能，促进企业提质增效。去库存方面，重点是深化户籍制度改革，加快农民工市民化，扩大有效需求；大力推进棚户区改造货币化安置和“以购代建”模式，力争货币化安置比例达到50%以上；进一步打通商品房市场与住房租赁市场、保障性住房的渠道，尽早把公租房扩大到非户籍人口，加快消化房地产库存，促进房地产业健康稳定发展。

加快重点领域改革。财税金融改革方面，稳步推进“营改增”，健全完善地方税体系；支持企业通过股票上市、股权交易、发行债券等方式融资，积极对接粤港澳金融资本，推进两广金融改革创新综合试验区建设。国资国企改革方面，以推进国有企业分类监管、政企分开和优化国资监管方式为切入点，加快市属国有企业重组整合与转型升级；推进国资监管体制改革，强化以管资本为主的国有资产监管工作，推进经营性国有资产集中统一监管工作；完善国资监管部门机构职能，加强监事会建设，提高履职能力。农村综合改革方面，继续推进农村土地承包经营权确权登记、现代特色农业（核心）示范区建设、长洲区农村集体资产股份权能改革等改革工作。统筹推进土地、科技、教育、卫生、社保等其他领域改革。

充分激发市场活力。深化行政审批制度改革，打造梧州版的“权力清单”、“责任清单”、“负面清单”。深化“一个窗口”受理、“一站式”审批，推进“串联”改“并联”审批。力争早日成立市行政审批局和行政执法局，实行“一枚公章管审批”“一支队伍管执法”。完善商事登记制度改革，全面落实 “先照后证”，巩固和扩大“三证合一”、“一照一码”改革成果。

（七）加大力度扩大开放合作。坚持向东开放战略，全方位、多角度打造粤桂深度合作的前沿阵地，不断提升我市开放合作水平。

深化区域战略合作。主动融入“一带一路”和粤桂黔高铁经济带，继续扩大面向粤港澳的开放合作，深化同广州、佛山、肇庆、云浮的战略合作，加快广佛肇梧一体化进程。以中国—东盟博览会为平台，扩大与东盟各国的合作交流。继续办好梧州宝石节和茶叶博览会等大型展会，进一步提高梧州在国内外的知名度。

加快粤桂合作特别试验区建设。贯彻落实《粤桂合作特别试验区建设实施方案》，建立健全体制机制，全力推动政策落地，大力推进道路、供电、供水、污水处理等基础设施建设。加快现代生产性服务业集聚区、两广金融改革创新综合试验区、中国—东盟环保技术和产业合作交流示范基地、粤桂合作企业总部基地、中小企业创业创新基地建设，争取国光电子、航天北斗超电容、丰冠风光能等重点项目部分建成投产。力争试验区工业总产值增长20%。

提高招商质量和水平。准确把握新常态下产业发展方向和产业转移趋势，围绕完善产业链条面向珠三角、长三角等地区精准招商，努力引进一批投资规模大、市场前景好、创税能力强的大项目。力争招商引资实际到位资金580亿元以上，建立健全招商引资激励约束机制，加强对招商引资签约数量和签约项目履约率、开工率、资金到位率、投产率等方面考核。

大力发展外向型经济。深入实施加工贸易倍增计划，大力开拓新兴市场，支持龙头企业扩大进出口业务，提升对外贸易水平和规模。做好梧州综合保税区申报工作，推进海关“无纸化”通关、口岸“单一窗口”建设，提高通关效率。运用“互联网+”，大力发展跨境电子商务等外贸新业态，培育壮大外贸新增长点。

（八）加大力度保障和改善民生。坚决保住基本民生，兜住民生底线，全力抓好事关人民群众福祉的民生实事，让人民群众共享发展成果。

推动创业扩大就业。大力推进大众创业、万众创新，降低创业创新成本，打造一批创业孵化基地、众创空间、农民工创业园。实施更加积极的就业政策，统筹做好零就业家庭、残疾人、低保对象、下岗失业人员和就业困难大学生等各类群体就业工作。落实好援企稳岗、社保补贴、税费减免等政策，力争城镇新增就业2.5万人，农村劳动力转移就业4.5万人次。

增强社会保障能力。实施全民参保登记计划，努力实现法定人群全覆盖。实现社会保障“一卡通”，推进“五险合一”征缴工作，确保各项社保待遇按时足额发放。推进城乡居民大病保险和新农合市级统筹工作，完善被征地农民社会保障政策，推进城乡社会救助体系建设。

抓好民生实事工程。筹措资金43亿元以上，重点抓好住房保障、创业就业、社会保障、医疗卫生、文化教育、便民服务等民生实事项目，让人民群众有更多的获得感和幸福感。

统筹发展社会事业。坚持教育优先发展战略，统筹安排5000万元支持学前教育发展，投入2.8亿元改善农村义务教育学校基本办学条件，筹措3000万元用于高中基础能力建设。支持做好梧州学院升格为大学相关工作，积极筹办梧州高等医学院校。启动健康梧州建设，创建西江生态养老产业示范区，加快医疗卫生基础设施建设步伐，全面推动公立医院综合改革和分级诊疗，着力推进区域卫生计生信息化平台建设，巩固和完善新农合制度。落实全面两孩政策。切实保障公众食品药品安全。加快西江文化博物馆、苍海文化中心、苍海科普基地、梧州粤剧保护与传承基地等项目建设，继续办好广西（梧州）粤剧节。筹建梧州市体校校舍，办好第十二届市运动会暨第二届全民健身运动会。完善应急管理体系，提高对气象、水文、地质、地震等自然灾害的监测预警和防御能力。推进国防教育、国防动员和后备力量建设，提升双拥工作水平。发展老龄事业，依法保障妇女儿童和残疾人的合法权益，支持工会、共青团、妇联等群团组织开展工作。统筹做好审计、统计、民族宗教、人防、编制、外事、侨务、对台、档案、地方志等工作。

全力维护社会稳定。深入推进“平安梧州”、“法治梧州”建设，加强社会治安防控体系建设，严厉打击各类违法犯罪，加大“扫黄打非”力度，启动“七五”普法工作，加快构建公共法律服务体系。强化网络舆情监控管理，提高舆论引导能力，传播社会正能量。加强重大决策社会稳定风险评估，畅通群众利益表达渠道。严格落实“党政同责、一岗双责、失职问责”的工作要求，强化企业安全主体责任落实，加强对重点场所、重点行业领域、重点企业的隐患排查整治，确保不发生重特大安全事故。

各位代表！新常态、新形势、新任务对政府工作提出了新的更高的要求，我们要以更加务实的作风，努力建设人民满意的政府。推进依法行政，全面履行政府职能，严格按照法定程序行使权力，加快建设法治政府。自觉接受人大及其常委会的监督，定期向人大及其常委会报告工作，支持人民政协履行政治协商、民主监督、参政议政职能，切实做好人大代表议案、建议和政协提案办理工作。认真听取各民主党派、工商联、无党派人士和各人民团体的意见建议。全面推进政务公开，推广电子政务和网上办事。严格执行重大行政决策法定程序，切实提高政府决策的科学化、民主化和法治化水平。严格落实党风廉政建设主体责任，坚定不移推进政府系统党风廉政建设和反腐败工作。

各位代表！加快发展，社会所需；小康愿景，民心所向。让我们紧密团结在以习近平同志为总书记的党中央周围，立足新起点，开启新征程，克难攻坚，真抓实干，锐意进取，为完成我市“三个实现”目标而努力奋斗！
